# Surfactant Additives Containing Hydrophobic Fluorocarbon Chains and Hydrophilic Sulfonate Anion for Highly Reversible Zn Anode

**DOI:** 10.3390/molecules28104177

**Published:** 2023-05-18

**Authors:** Jinxian Huang, Zhao Fu, Chuan-Fu Sun, Wenzhuo Deng

**Affiliations:** 1 College of Chemistry, Fuzhou University, Fuzhou 350108, China; huangjinxian@fjirsm.ac.cn (J.H.); fuzhao@fjirsm.ac.cn (Z.F.); 2 CAS Key Laboratory of Design and Assembly of Functional Nanostructures, Fujian Key Laboratory of Nanomaterials, and State Key Laboratory of Structural Chemistry, Fujian Institute of Research on the Structure of Matter, Chinese Academy of Sciences, Fuzhou 350002, China; cfsun@fjirsm.ac.cn

**Keywords:** Zn anode, Zn dendrite, side reaction, electrolyte additive, potassium perfluoro-1-butanesulfonate

## Abstract

Aqueous zinc-ion batteries (AZIBs) show enormous potential as a large-scale energy storage technique. However, the growth of Zn dendrites and serious side reactions occurring at the Zn anode hinder the practical application of AZIBs. For the first time, we reported a fluorine-containing surfactant, i.e., potassium perfluoro-1-butanesulfonate (PPFBS), as an additive to the 2 M ZnSO_4_ electrolyte. Benefitting from its hydrophilic sulfonate anion and hydrophobic long fluorocarbon chain, PPFBS can promote the uniform distribution of Zn^2+^ flux at the anode/electrolyte interface, allowing the Zn/Zn cell to cycle for 2200 h. Furthermore, PPFBS could inhibit side reactions due to the existence of the perfluorobutyl sulfonate (C_4_F_9_SO_3_^−^) adsorption layer and the presence of C_4_F_9_SO_3_^−^ in the solvation structure of Zn^2+^. The former can reduce the amount of H_2_O molecules and SO_4_^2−^ in contact with the Zn anode and C_4_F_9_SO_3_^−^ entering the Zn^2+^-solvation structure by replacing SO_4_^2−^. The Zn/Cu cell exhibits a superior average CE of 99.47% over 500 cycles. When coupled with the V_2_O_5_ cathode, the full cell shows impressive cycle stability. This work provides a simple, effective, and economical solution to the common issues of the Zn anode.

## 1. Introduction

The dwindling reserves of fossil fuels and growing environmental awareness are driving the continued research on reliable large-scale battery systems for the storage and utilization of intermittent-generated renewable energy [1,2,3,4,5]. Lithium-ion batteries (LIBs) are the most extensively studied rechargeable batteries; however, they are not suitable for large-scale energy storage due to the scarcity of lithium resources and safety concerns [6,7]. Rechargeable AZIBs have been identified as an exceptionally promising solution owing to the intrinsic properties of the metal Zn anode, including a high theoretical capacity (820 mA h g^−1^ and 5854 mA h cm^−3^), low costs, the inherent safety of the aqueous electrolytes, environmental benignity, and easy production [8,9,10,11]. Despite the multiple advantages, Zn dendrites and side reactions obstruct the practical application of AZIBs. The Zn dendrites are mainly caused by the uneven flux of Zn^2+^ ions, which can pierce the separator and reach the cathode, eventually causing the battery to short-circuit. Moreover, the Zn dendrite enlarges the volume of the Zn anode, which increases the sites of side reactions. These side reactions are mainly initiated by the decomposition of H_2_O molecules on the Zn anode surface, resulting in H_2_ evolution and OH^−^ generation. The continuous accumulation of OH^−^ at the Zn anode can induce the coalescence of Zn^2+^ and other electrolyte components (e.g., SO_4_^2−^) to form insulating side products (e.g., Zn_4_SO_4_(OH)_6_·4H_2_O), reducing the Coulombic efficiency (CE) [12]. In addition, these insulating byproducts further impede the homogeneous Zn plating. Consequently, the homogeneous flux of Zn^2+^ and the elimination of side reactions are critical to achieve a long lifespan and a high CE for AZIBs.

Recently, a variety of approaches have been developed to promote uniform Zn deposition and inhibit the side reactions. Among them, interface modification [13,14,15,16,17,18], separator optimization [19,20,21,22,23,24], and substrate design [25,26,27,28] are effective ways to improve the lifespan of the battery, but the complex preparation process limits their application. Easily produced super-concentrated electrolytes [29,30,31,32,33] bearing a low water level exhibit a special Zn^2+^-solvation structure, favoring the uniform deposition of Zn and suppressing side reactions. However, the relatively low ionic conductivity leads to poor rate performance and the high salt concentration increases the cost. Alternatively, electrolyte additives in dilute solutions [34,35,36,37,38,39,40,41,42,43] are another feasible solution. However, there is only a narrow range of choices of electrolyte additives, and more need to be explored to further lower the cost and provide better performance.

A water-soluble fluorinated surfactant is an effective electrolyte additive, which can benefit the formation of uniform Zn^2+^ flux by reducing the contact angle of the Zn anode/electrolyte interface, thereby promoting smooth and dense Zn deposition. Due to the high electron affinity (328 kJ mol^−1^) and electronegativity (3.98) of fluorine, and the low polarizability (α = 0.557 × 10^−24^ cm^3^), the long fluorocarbon chains with more than four -CF_2_- groups have a good hydrophobic effect [44]. Parkin et al. showed that the addition of 10 mM perfluorooctanoic acid (PFOA) additive containing a -CF_2_- group in 3 M ZnSO_4_ electrolyte was able to inhibit the growth of Zn dendrites and improve the cycle stability of the battery [42]. However, fluorinated surfactants with seven or more -CF_2_- groups, such as perfluorooctane sulfonates (PFOS) and perfluorooctanoic acid (PFOA), would lead to bioaccumulation in animals and humans, causing serious health and environmental problems [44]. Guo et al. stated that adding 1 mg m L^−1^ sodium dodecyl benzene sulfonate (SDBS) additive containing a strongly hydrophilic -SO_3_ anion to 1 M Zn(SO_3_CF_3_)_2_ and 1 M Li(SO_3_CF_3_) electrolyte can also effectively improve the battery life [45]. Therefore, fluorinated surfactants containing a -SO_3_ anion and an appropriate amount of -CF_2_- groups may be a good choice for the large-scale energy storage of AZIBs due to their moderate surface activity, water solubility, biosafety, and environmental friendliness.

In this work, we introduced a PPFBS additive containing one -SO_3_ anion and four -CF_2_- groups into a 2 M ZnSO_4_ electrolyte and studied its performance in AZIBs. The results show that C_4_F_9_SO_3_^−^ can be adsorbed on the Zn anode surface, reducing the contact angle between the electrolyte droplet and the Zn anode surface (83° to 59°). The hydrophobic and strongly electronegative C_4_F_9_SO_3_^−^ adsorption layer on the Zn anode surface diminishes the interactions between H_2_O molecules and the Zn anode, thereby inhibiting side reactions. In addition, molecular dynamics (MD) simulations show that C_4_F_9_SO_3_^−^ can participate in the Zn^2+^-solvation structure by substituting SO_4_^2−^ and reducing the Zn_4_SO_4_(OH)_6_·H_2_O. Furthermore, the Zn/Zn cell cycles for 2200 h at a current density of 5 mA cm^−2^ with a capacity of 1 mAh cm^−2^, and the Zn/Cu cell shows a high average CE of 99.47% over 500 cycles at a current density of 2 mA cm^−2^, with a capacity of 1 mAh cm^−2^. Finally, the cycle stability and rate performance of the Zn/V_2_O_5_ battery are both improved.

## 2. Results and Discussion

**Figure 1:**
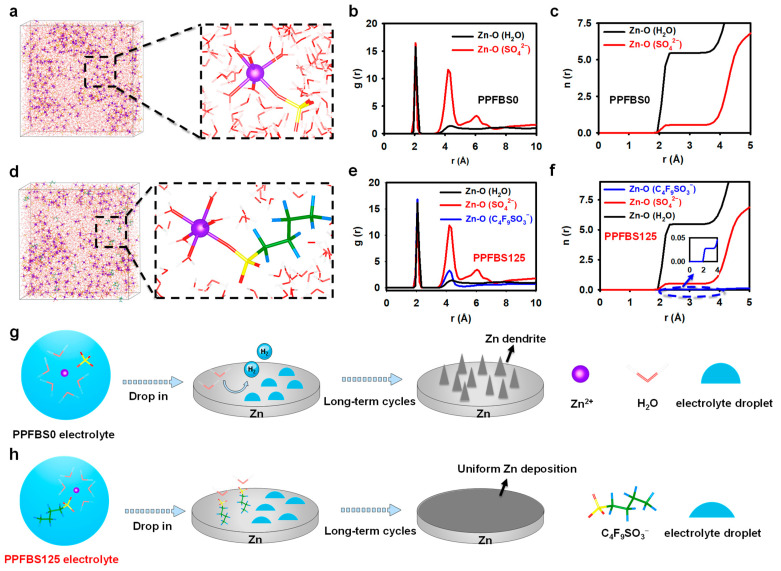
Snapshots of the molecular dynamics (MD) simulation models and an enlarged view of the Zn^2+^-solvation structure in the electrolytes with (**a**) PPFBS0 and (**d**) PPFBS125 electrolytes; radial distribution functions (RDFs) obtained from MD simulations for (**b**) PPFBS0 and (**e**) PPFBS125 electrolytes; coordination number (CN) obtained from MD simulations for (**c**) PPFBS0 and (**f**) PPFBS125 electrolytes; schematic illustration of the Zn^2+^-solvation structure and corresponding deposition behavior in the electrolytes with (**g**) PPFBS0 and (**h**) PPFBS125 electrolytes.

As a surfactant, PPFBS can reach the interface by the low energy barrier method and adsorb on the interface through intermolecular interaction. Specifically, the hydrophilic sulfonic head groups of hydrophilic surfactant PPFBS may be orientated towards the electrolyte, while the hydrophobic perfluoro butyl tail groups may be in contact with the Zn anode surface. Under the synergistic effect of these two groups, PPFBS can be separated and self-assembled near the Zn anode/electrolyte interface to form a solid and orderly arranged adsorption layer. Energy-dispersive spectroscopy (EDS) was used to confirm the adsorption of PPFBS. The carbon, fluorine, potassium, and sulfur signals on the surface of the Zn plate after immersion in a 0.125 M PPFBS solution were rinsed with flowing water and were detected and their even distribution was revealed (Figure S1). The hydrophobic C_4_F_9_SO_3_^−^ adsorption layer may give rise to additional energy barriers preventing the solvent and anions from reaching the Zn anode surface. We conducted MD simulations to investigate the influence of the addition of C_4_F_9_SO_3_^−^ on the coordination environment of the Zn^2+^ ion. Figure 1a,d show a snapshot of the MD 3D model and an enlarged view of the Zn^2+^-solvation structure in the two electrolytes. For the 2 M ZnSO_4_ electrolyte (PPFBS0 electrolyte), the first solvation shell of the Zn^2+^ ion is composed of one SO_4_^2−^ anion and five H_2_O molecules, whereas for the other, five H_2_O molecules along with one C_4_F_9_SO_3_^−^ comprise the first solvation shell of Zn^2+^ for the 2M ZnSO_4_ electrolyte containing 125 mM PPFBS (PPFBS125 electrolyte). Figure 1b,e depict the corresponding radial distribution functions (RDFs) of the two electrolytes. The RDFs show a sharp peak located at around 2.06 Å for the PPFBS0 electrolyte, indicative of an averaged Zn-O (H_2_O) distance. Apart from the contribution of SO_4_^2−^, water plays a significant role in the solvation shell. As for the PPFBS125 electrolyte, the Zn-O (C_4_F_9_SO_3_^−^) peak overlaps the Zn-O (H_2_O) peak, verifying the participation of C_4_F_9_SO_3_^−^ in the solvation structure of Zn^2+^ ions. The coordination number (CN) of the two electrolytes shows the proportion of each ion in the first solvation shell of Zn^2+^ (Figure 1c,f). The CN results show that the average number of SO_4_^2−^ around each Zn^2+^ decreases from 0.53 to 0.50, and the number of C_4_F_9_SO_3_^−^ increases from 0 to 0.03 upon the addition of 125 M PPFBS, which may help to reduce the SO_4_^2−^-involved byproducts during Zn^2+^ deposition (Figure S2). A schematic illustration of the Zn^2+^-solvation structure and the corresponding deposition behavior is shown in Figure 1g,h.

**Figure 2:**
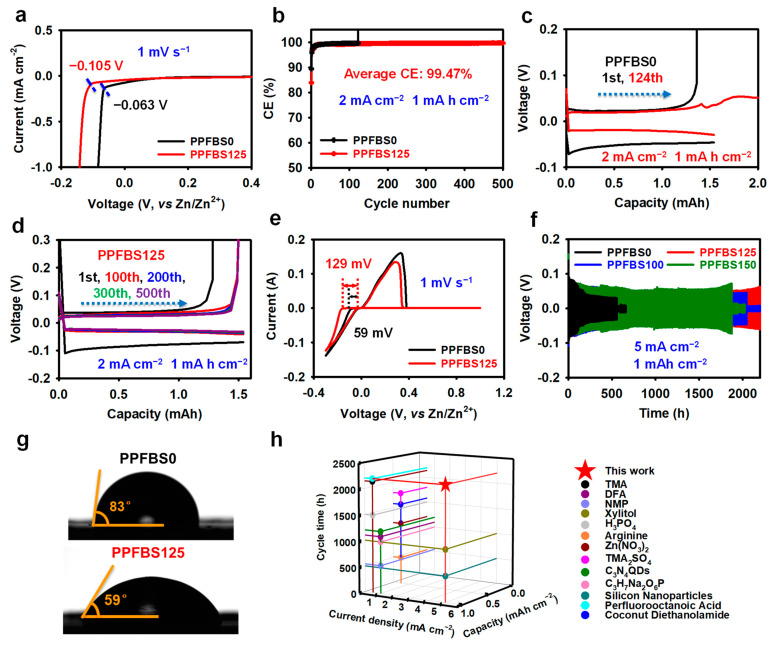
(**a**) Linear sweep voltammetry (LSV) characterization in the different electrolytes; (**b**) Coulombic efficiency (CE) of Zn/Cu cells in the different electrolytes; voltage profiles of Zn/Cu cells using (**c**) PPFBS0 and (**d**) PPFBS125 electrolytes at various cycles; (**e**) cyclic voltammogram (CV) profiles of Zn/Cu cells in the different electrolytes; (**f**) cycling stability of Zn/Zn cells in the various electrolytes; (**g**) the contact angle on Zn substrates using different electrolytes; (**h**) comparison of the electrochemical performance of electrolyte additives in Zn/Zn cells.

Linear sweep voltammetry (LSV) was used to evaluate the effect of the PPFBS additive on the electrochemical stability of the electrolyte (Figure 2a). The starting hydrogen evolution potential of the PPFBS125 electrolyte is −0.105 V, which is lower than that of the PPFBS0 electrolyte (−0.063V), which verifies that the C_4_F_9_SO_3_^−^ adsorption layer can prevent the contact between H_2_O molecules and the Zn anode, thereby restraining the decomposition of H_2_O molecules. To further study the effect of PPFBS additives on side reactions, the Zn/Cu cell was assembled and tested at a current density of 2 mA cm^–2^ with a capacity of 1 mA h cm^–2^. The Zn/Cu cell with the PPFBS0 electrolyte showed an inferior average CE of 99.22%, and the battery failed at the 124th cycle (Figure 2b,c). In the Zn/Cu cell with the PPFBS125 electrolyte, the cell achieves a longer cycle life of 500 cycles and a higher average CE of 99.47% (Figure 2b,d). The better CE performance originates from the reduction of side reactions, and the extension of the cycle life is mainly due to the PPFBS additive inhibiting the growth of Zn dendrites induced by uneven Zn deposition.

The contact angle between the electrolyte droplet and the Zn metal anode may give information on surface tension. Figure 2g displays a contact angle of 59° for the PPFBS125 electrolyte, which is significantly smaller than that of the PPFBS0 counterpart (85°), which may be the synergetic effect of the super-hydrophobic long fluorocarbon chain and the hydrophilic sulfonate anion of PPFBS. The increased wettability of the electrolyte on the Zn anode may promote the uniform distribution of the Zn^2+^ flux, thereby facilitating the homogeneous plating of Zn. Even Zn deposition usually means that there is a small critical Zn nucleus radius brought by a large initial nucleation overpotential. The cyclic voltammogram (CV) curves of Zn/Cu cells are used to evaluate the initial nucleation overpotential. As indicated in Figure 2e, the initial nucleation overpotential of Zn increases from 59 m V to 129 m V when the PPFBS additive is applied, which further proves the impact of PPFBS on Zn deposition. The Zn/Zn cell was used to investigate the effect of Zn deposition on battery life. The Zn/Zn cell without PPFBS additives can only cycle for 484 h at a current density of 5 mA cm^−2^ with a capacity of 1 mA h cm^−2^, while the Zn/Zn cell with 100 mM, 125 mM, and 150 mM PPFBS additives can operate for 2042 h, 2200 h, and 1872 h, respectively (Figure 2f). This performance is superior to that of most of the reported electrolyte additives (Figure 2h, details in Table S1, Supporting Information).

**Figure 3:**
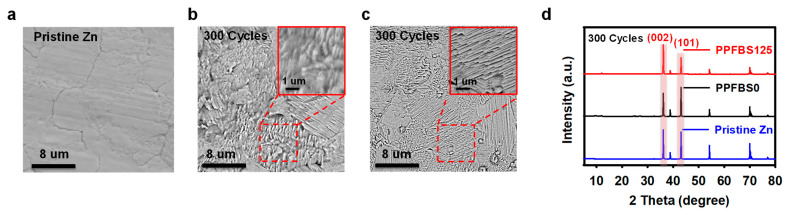
(**a**) Top-view scanning electron microscopy (SEM) images of the pristine Zn plate; SEM images of the Zn anode surface from Zn/Zn cells using (**b**) PPFBS0 and (**c**) PPFBS125 electrolytes after 300 cycles; (**d**) X-ray diffraction (XRD) patterns of pristine Zn plate and Zn electrodes from Zn/Zn cells using different electrolytes after 300 cycles.

Scanning electron microscopy (SEM) was used to observe the morphology of the Zn electrodes after cycling. As presented in Figure 3a, the surface of the pristine Zn plate is uniform. After 300 cycles at a current density of 5 mA cm^−2^ and a capacity of 1 mA h cm^−2^, the Zn anode of the cell adopting the PPFBS125 electrolyte shows a layered and ordered structure, while the vertical Zn dendrites were detected in the cell without PPFBS additive (Figure 3b,c). The optical images of the pristine Zn plate and the Zn anode after 500 cycles in two different electrolytes were further tested (Figure S3). The optical images of the pristine Zn plate show a smooth surface. After 500 cycles at 5 mA cm^−2^ and 1 mA h cm^−2^, the optical images show that the Zn anode surface is covered with a thick layer of Zn and some byproducts in the PPFBS0 electrolyte. In the PPFBS125 electrolyte, the Zn deposition and byproduct layer on the surface of the Zn anode is significantly thinner, and its morphology is similar to that of the pristine Zn plate. This result confirms that the PPFBS electrolyte additive can promote uniform Zn deposition and hinder side reactions. To further evaluate the effect of PPFBS electrolyte additives on the growth of Zn dendrites, the top-view SEM images and cross-section SEM images of Zn anodes after 500 cycles in PPFBS0 electrolyte and PPFBS125 electrolyte were obtained (Figures S4 and S5). The top-view SEM images showed that the Zn anode surface was porous and fully covered with vertical Zn dendrites using the PPFBS0 electrolyte, while the Zn surface was flat using the PPFBS125 electrolyte. The cross-section SEM images of the Zn anode indicate that in the PPFBS0 electrolyte, the Zn anode surface is covered with sharp Zn dendrites. These Zn dendrites can not only pierce the separator and cause the short-circuit of the battery but also can fall off from the surface of the Zn anode, reduce the amount of reversible Zn^2+^, and rapidly decay the specific capacity of the battery [45]. In the PPFBS125 electrolyte, the surface of the Zn anode is uniform and smooth. This is consistent with the results observed in the top-view SEM images.

X-ray diffraction (XRD) was used to further assess the effect of PPFBS electrolyte additives on the morphological evolution of the Zn anode in different electrolytes (Figure 3d). After 300 cycles at 5 mA cm^−2^ and 1 mA h cm^−2^, all the XRD characteristic peaks of the Zn anode using the PPFBS125 electrolyte are attributed to Zn, indicating that no SEI films were formed on the surface of the Zn anode, and the additive is inert in the battery. In addition, apparently, the preferred orientation of the (002) crystal plane occurred during the deposition of Zn when the PPFBS125 electrolyte was applied. This phenomenon has been reported to be beneficial to the uniform plating of Zn. Archer et al. used a low lattice mismatch graphene layer to guide Zn^2+^ deposition along the (002) crystal plane [16], and they showed that Zn exhibited a strong tendency to deposit as platelets on the surface of the graphene layer, thus effectively impeding Zn dendrite growth and side reactions [16]. Liang et al. produced a Zn plate with preferentially exposed (002) crystal planes by the large rolling deformation method for aqueous ZIB [46]. Their results indicate that the even interfacial charge density and stronger adsorption energies in the parallel direction rather than the vertical direction of the (002) crystal plane promoted the uniform Zn deposition and inhibited the growth of Zn dendrites [46]. Moreover, owing to the strong catching ability of Zn atoms and the high free energy for hydrogen evolution of the (002) crystal plane, the side reactions are also effectively suppressed [46]. In contrast, for the battery using the PPFBS0 electrolyte, Zn is mainly plated along the (101) plane. The inclined stacking of the (101) surface is more likely to induce the vertical growth of Zn dendrites and side reactions. This result further proves that PPFBS is conducive to uniform and dense Zn deposition and the prevention of side reactions, thereby improving the cycle stability of the battery.

**Figure 4:**
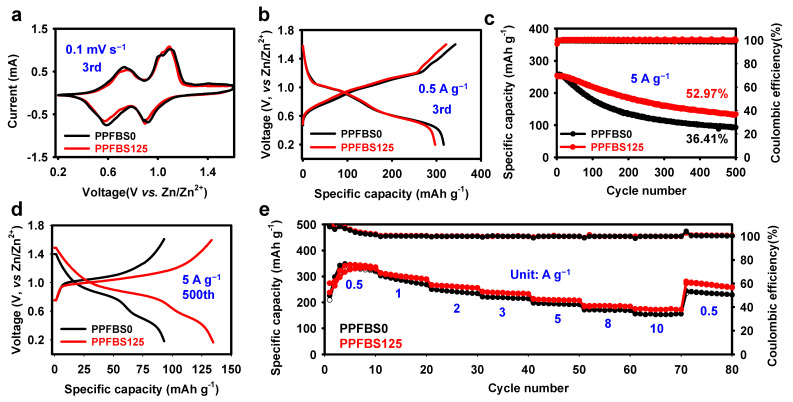
(**a**) CV profiles of the Zn/V_2_O_5_ cells at 3rd cycle with a scan rate of 0.1 mV s^−1^; (**b**) galvanostatic charge–discharge profile of the Zn/V_2_O_5_ cells at 3rd cycle with a current density of 500 mA g^−1^; (**c**) long-term cycling performance and CE of the Zn/V_2_O_5_ cells in the different electrolytes; (**d**) galvanostatic charge–discharge profile of the Zn/V_2_O_5_ cells at 500th cycle with a current density of 5 A g^−1^; (**e**) rate performance of the Zn/V_2_O_5_ cells in different electrolytes.

The V_2_O_5_ was synthesized to evaluate the effect of PPFBS additives on the electrochemical performance of cathode materials. As shown, all XRD diffraction peaks can be indexed to the standard V_2_O_5_ (PDF # 41-1426) and SEM images show that the material is nanoparticles (Figures S6 and S7). It can be seen from the EDS results that the V and O elements are evenly distributed in the sample (Figure S8). The CV curves and charge–discharge curves of Zn/V_2_O_5_ cells were further tested in two electrolytes. As shown in Figure 4a, both CV curves have two pairs of redox peaks. In addition, the charge–discharge curves display that the Zn/V_2_O_5_ cells have the same charge–discharge platform in the two electrolytes, indicating that PPFBS has no effects on the redox reaction (Figure 4b). The cycling stability of Zn/V_2_O_5_ batteries was examined at a current density of 5 A g^–1^ (Figure 4c,d). The capacity retention increased from 36.41% to 52.97% in 500 cycles when the 125 mM PPFBS was added. Furthermore, the rate performance of the Zn/V_2_O_5_ cells in the different electrolytes is presented in Figure 4e. The Zn/V_2_O_5_ cell using the PPFBS125 electrolyte displays specific capacities of 326.3, 299.7, 264.0, 235.1, 209.7, 184.4, and 172.2 mA h g^−1^ at 0.5, 1, 2, 3, 5, 8, and 10 A g^−1^, respectively (Figure 4e). In comparison, the PPFBS0 electrolyte has specific capacities of 329.3, 285.6, 235.0, 215.9, 193.0, 168.9, and 152.8 mA h g^−1^ at 0.5, 1, 2, 3, 5, 8, and 10 A g^−1^, respectively. Corresponding galvanostatic charge–discharge profiles are displayed in Figure S9. The improved performance because of the addition of PPFBS stems from the suppression of side reactions and dendrite growth.

## 3. Materials and Methods

### 3.1. Materials

ZnSO_4_⋅7H_2_O (AR, Sinopharm Chemical Reagent Co., Ltd., Shanghai, China), V_2_O_5_ (AR, Shanghai Macklin Biochemical Co., Ltd., Shanghai, China), potassium perfluoro-1-butanesulfonate (PPFBS, 97%, Aladdin Chemical Reagent Co., Ltd., Shanghai, China), ethylene glycol (AR, Sinopharm Chemical Reagent Co., Ltd., Shanghai, China), N-methyl-2-pyrrolidone (NMP, Aladdin, AR), polyvinylidene difluoride (PVDF, 99%, Hefei Kejing Materials Technology Co., Ltd., Hefei, China), Super-P carbon black (99.8%, Hefei Kejing Materials Technology Co., Ltd., Hefei, China). All chemicals and reagents were used directly, without further processing.

### 3.2. Synthesis of Electrolytes

The 2 M ZnSO_4_ aqueous electrolyte was prepared using ZnSO_4_·7H_2_O and deionized water based on the molar ratio. The PPFBS-containing electrolytes were synthesized by dissolving a stoichiometric concentration of PPFBS (range 100 to 150 mM) in the as-prepared ZnSO_4_ electrolytes.

### 3.3. Synthesis of V_2_O_5_ Cathode Material

First, 10 mM V_2_O_5_ was added into a solution containing 30 mL of deionized water and 20 g of ethylene glycol under vigorous stirring to obtain a uniform solution. The resulting mixture was transferred to a 100 mL autoclave, heated at 200 °C for 24 h, and then cooled to room temperature. The collected products were centrifuged, washed, and dried at 80 °C for 12 h. The obtained precipitates were annealed at 330 °C for 30 min with a heating rate of 2 °C min^−1^. V_2_O_5_ powders were produced after cooling to room temperature [35].

### 3.4. Synthesis of the V_2_O_5_ Composite Electrode

The V_2_O_5_ powder was mixed with Super-P carbon black and PVDF with a mass ratio of 70:20:10 in NMP to obtain a slurry. The as-prepared slurry was cast on carbon cloth and dried under a vacuum at 80 °C for 12 h to obtain the V_2_O_5_ electrode.

### 3.5. Electrochemical Evaluation

CR2032-type coin cells with different aqueous electrolytes were assembled under an ambient environment. NEWARE battery testing systems (Neware Technology Co., Ltd., Shenzhen, China) were used to conduct battery performance tests for cycle stability, and an electrochemical workstation (CHI 760D, Shanghai Chenhua Instrument Co., Ltd., Shanghai, China) was employed to analyze other electrochemical behavior, such as LSV.

### 3.6. Details of Molecular Dynamics (MD) Simulation

The aqueous electrolyte models were constructed with the aid of the PACKMOL program [47]. All systems were represented by cubic boxes containing 400 Zn^2+^ ions and corresponding species in different ratios; the original unit cell edges were fixed at 80 Å. Initial configurations were relaxed to minimize total energies and remove nonreasonable physical atom contacts prior to isothermal–isobaric (NPT) molecular dynamics (MD) computations. We employed the Monte Carlo barostat to keep the pressure at a constant value of 1 bar, and the Langevin thermostat was used to control the temperature at a level of 298.15 K. The Lorentz-Berthelot combing rules, i.e., σij = (σii + σjj)/2 and εij = (εii × εjj)/2, were applied for the non-bonded pairwise Lennard–Jones interactions. The Coulombic electrostatic interactions were calculated based on the PME method. A total run of 15 ns was performed with a time step of 1 fs. We exploited the TIP3P [48] model for water and the CHARMM [49] force field for divalent Zn^2+^ ions, and we took the reported force field parameters for K^+^ monovalent cations [50], SO_4_^2−^ ion [51], and C_4_F_9_SO_3_^−^ ion [52]. The OpenMM 7 code [53] was adopted for all the MD runs. The trajectories were recorded at 1 frame per ps, the last 1 ns of which was processed using the MDAnalysis package [54]; information pertaining to radial distribution functions (RDFs) and coordination numbers (CN) was thus extracted.

## 4. Conclusions

In this work, PPFBS additive was added into the 2M ZnSO_4_ electrolyte to suppress side reactions and regulate Zn deposition. The formed C_4_F_9_SO_3_^−^ adsorption layer separates H_2_O molecules and SO_4_^2−^ from the Zn anode, and thus inhibits the hydrogen evolution and reduces the formation of byproducts. MD simulations confirmed the presence of C_4_F_9_SO_3_^−^ in the Zn^2+^-solvation structure. The assembled Zn/Cu cell with the C_4_F_9_SO_3_^−^ bearing electrolyte exhibits a superior average CE of 99.47% over 500 cycles at 2 mA cm^−2^ with a capacity of 1 mAh cm^−2^. Moreover, the trace amount of PPFBS forms a stable, highly surface-activated adsorption layer. This adsorption layer reduces the contact angle at the interface (from 83° to 59°), which facilitates the formation of uniform Zn^2+^ flux and inhibits the growth of Zn dendrites. The Zn/Zn cell cycles over 2200 h at 5 mA cm^−2^ with a capacity of 1 mAh cm^−2^. After cycling, the Zn anode exhibits a preferential orientation of the (002) plane. Furthermore, PPFBS improves the cycle stability and rate performance of the Zn/V_2_O_5_ battery. This effective and low-cost PPFBS additive is expected to play a more important role in the development of better electrolyte formulas for AZIBs.

## Data Availability

Not applicable.

## Supplementary Materials

The following supporting information can be downloaded at: https://www.mdpi.com/article/10.3390/molecules28104177/s1. Figure S1: EDS mapping spectra of Zn plate after soaking in 0.125 M PPFBS solution and rinsing with flowing water. Figure S2: (a) The average number of SO_4_^2−^ around each Zn^2+^ in the two electrolytes. (b) The average number of C_4_F_9_SO_3_^−^ around each Zn^2+^ in PPFBS125 electrolytes. Table S1: Comparison of the electrochemical performance of electrolyte additives in Zn/Zn symmetric cells. Figure S3: (a) Optical images of the pristine Zn plate; optical images of the Zn anode surface from Zn/Zn cells using (b) PPFBS0 and (c) PPFBS125 electrolytes after 500 cycles. Figure S4: Top-view SEM images of the Zn anode surface from Zn/Zn cells using (a,b) PPFBS0 and (c,d) PPFBS125 electrolytes after 500 cycles. Figure S5: Cross-section SEM images of the Zn anode surface from Zn/Zn cells using (a) PPFBS0 and (b) PPFBS125 electrolytes after 500 cycles. Figure S6: XRD patterns of V_2_O_5_. Figure S7: SEM image of V_2_O_5_. Figure S8: EDS mapping spectra of V_2_O_5_. Figure S9: Galvanostatic charge–discharge profile under different rates using (a) PPFBS0 and (b) PPFBS125 electrolytes. References [36,37,38,42,55,56,57,58,59,60,61,62,63] are cited in Supplementary Materials.

## Sample Availability

Samples of the compounds are available from the authors.
