# Supplementary material for: Surfactant Additives Containing Hydrophobic Fluorocarbon Chains and Hydrophilic Sulfonate Anion for Highly Reversible Zn Anode
Source: Molecules. 2023 May 18;28(10):4177. doi: 10.3390/molecules28104177 (PMC10221768; doi:10.3390/molecules28104177)
Supplement: Supplementary file 1 [file molecules-28-04177-s001.zip › molecules-2361091-supplementary.pdf]

Supplementary materials

Surfactant Additives Containing Hydrophobic Fluorocarbon Chains and Hydrophilic Sulfonate Anion for Highly Reversible Zn Anode

Jinxian Huang [1,2], Zhao Fu [1,2], Chuan-Fu Sun [2] and Wenzhuo Deng [2,*]

[1] College of Chemistry, Fuzhou University, Fuzhou 350108, China
[2] CAS Key Laboratory of Design and Assembly of Functional Nanostructures, Fujian Key Laboratory of Nanomaterials, and State Key Laboratory of Structural Chemistry, Fujian Institute of Research on the Structure of Matter, Chinese Academy of Sciences, Fuzhou 350002, China
* Correspondence: wzdeng@fjirsm.ac.cn

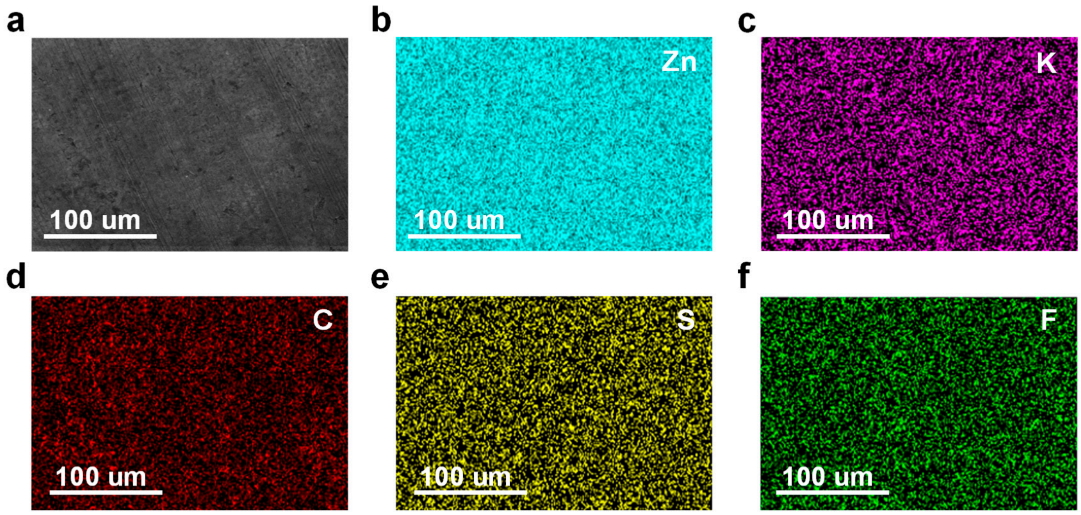

Figure S1. EDS mapping spectra of Zn plate after soaking in 0.125M PPFBS solution and rinsing with flowing water.

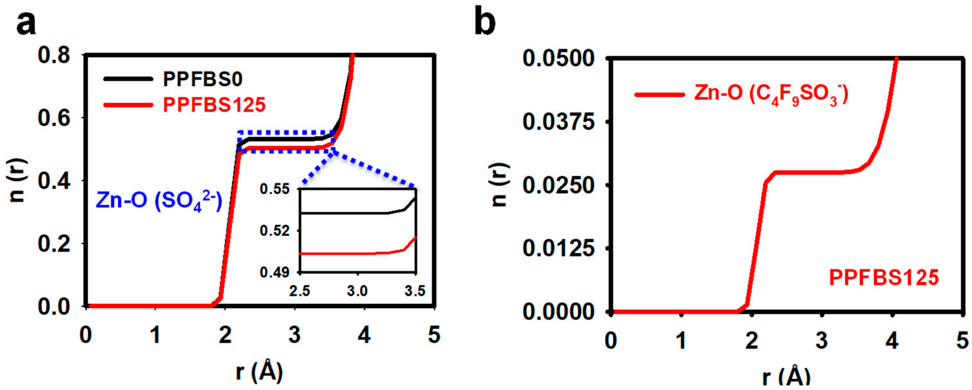

Figure S2. (a) The average number of SO_4^{2-} around each Zn^{2+} in the two electrolytes; (b) The average number of $C_4F_9SO_3^-$ around each Zn^{2+} in PPFBS125 electrolytes.

Table S1. Comparison of the electrochemical performance of electrolyte additives in Zn/Zn symmetric cells.

Additives	Current density ($mA\ cm^{-2}$)	Capacity ($mAh\ cm^{-2}$)	Cycle time	Reference
PPFBS	5	1	2200 h	This work
TMA	1	0.5	2145 h	[1]
DFA	1	1	1100 h	[2]
NMP	1	1	540 h	[3]
Xylitol	5	1	1000 h	[4]
H_3PO_4	1	0.5	1500 h	[5]
Arginine	0.5	0.5	515 h	[6]
$Zn(NO_3)_2$	0.5	0.5	1200 h	[7]
TMA_2SO_4	0.5	0.5	1800 h	[8]
C_3N_4QDs	1	1	1200 h	[9]
$C_3H_7Na_2O_6P$	1	1	1100 h	[10]
Silicon Nanoparticles	5	1	500 h	[11]
Perfluorooctanoic Acid	1	0.5	2200 h	[12]
Coconut Diethanolamide	0.5	0.5	1580 h	[13]

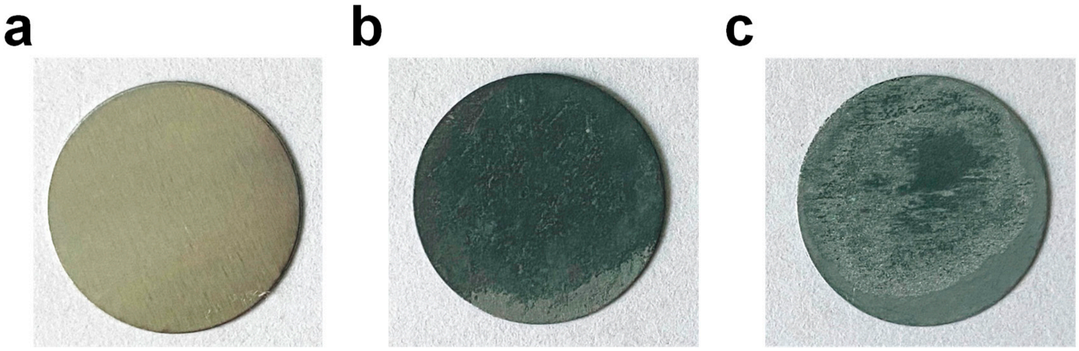

Figure S3. (a) Optical images of the pristine Zn plate; Optical images of the Zn anode surface from Zn/Zn cells using (b) PPFBS0 and (c) PPFBS125 electrolytes after 500 cycles.

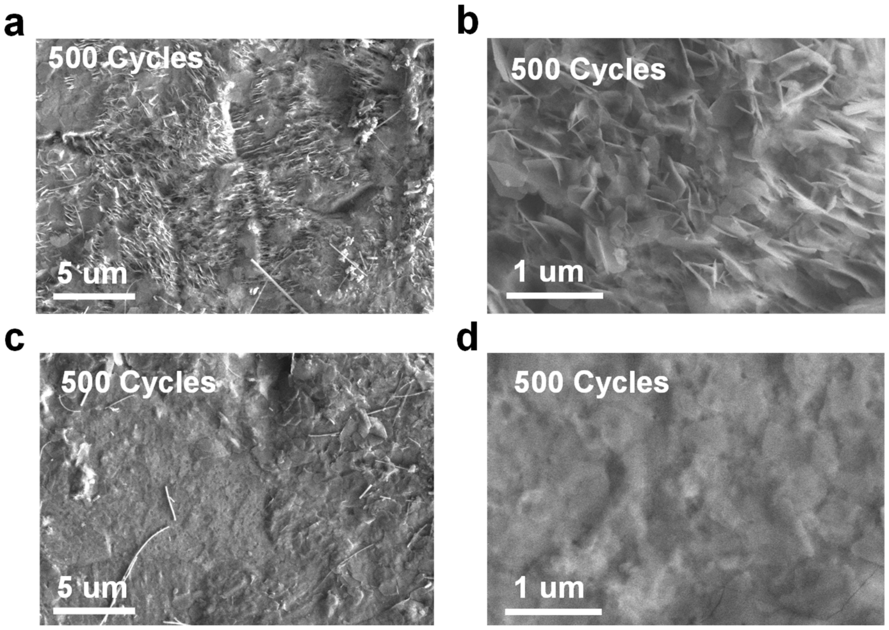

Figure S4. Top-view SEM images of the Zn anode surface from Zn/Zn cells using (a-b) PPFBS0 and (c-d) PPFBS125 electrolytes after 500 cycles.

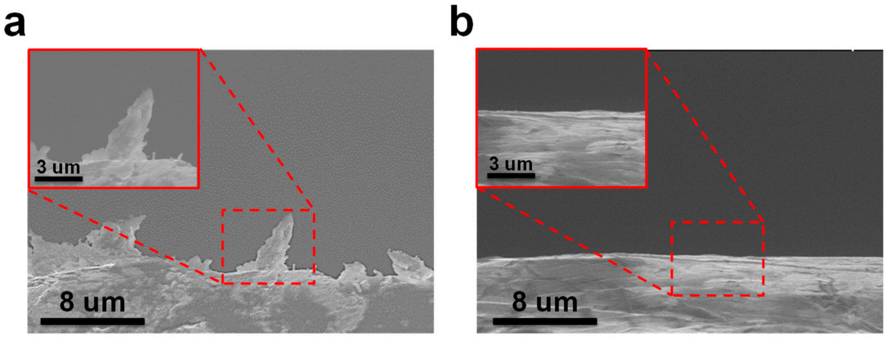

Figure S5. Cross-section SEM images of the Zn anode surface from Zn/Zn cells using (a) PPFBS0 and (b) PPFBS125 electrolytes after 500 cycles.

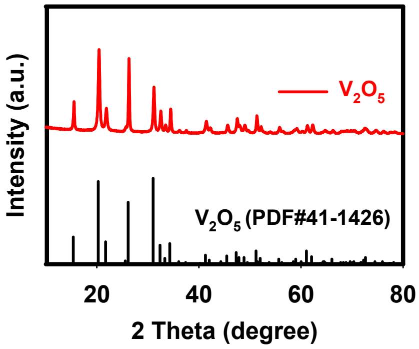

Figure S6. XRD patterns of V_2O_5.

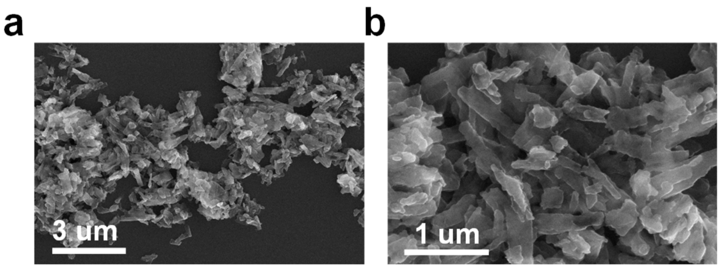

Figure S7. SEM image of V_2O_5.

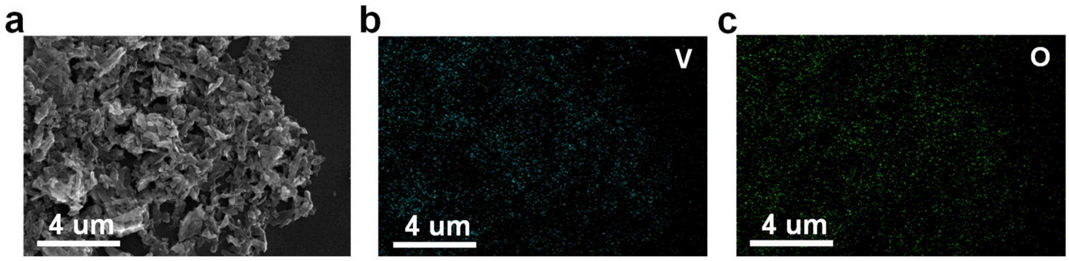

Figure S8. EDS mapping spectra of V_2O_5.

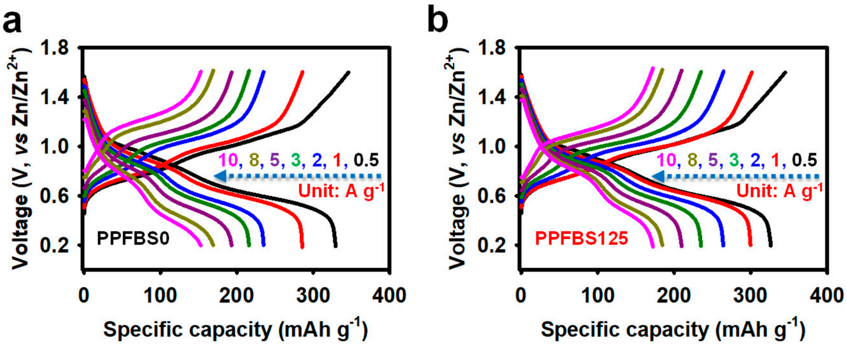

Figure S9. Galvanostatic charge-discharge profile under different rates using (e) PPFBS0 and (f) PPFBS125 electrolytes.

References

1. Yao, R.; Qian, L.; Sui, Y.; Zhao, G.; Guo, R.; Hu, S.; Liu, P.; Zhu, H.; Wang, F.; Zhi, C.; et al. A versatile cation additive enabled highly reversible zinc metal anode. *Adv. Mater.* **2022**, *12*, 2102780.
2. Tian, H.; Yang, J.-L.; Deng, Y.; Tang, W.; Liu, R.; Xu, C.; Han, P.; Fan, H.J. Steel anti-corrosion strategy enables long-cycle Zn anode. *Adv. Energy Mater.* **2022**, *13*, 2202603.
3. Li, T.C.; Lim, Y.; Li, X.L.; Luo, S.; Lin, C.; Fang, D.; Xia, S.; Wang, Y.; Yang, H.Y. A universal additive strategy to reshape electrolyte solvation structure toward reversible zn storage. *Adv. Energy Mater.* **2022**, *12*, 2103231.
4. Wang, H.; Ye, W.; Yin, B.; Wang, K.; Riaz, M.S.; Xie, B.-B.; Zhong, Y.; Hu, Y. Modulating cation migration and deposition with xylitol additive and oriented reconstruction of hydrogen bonds for stable zinc anodes. *Angew. Chem. Int. Ed.* **2023**, *135*, e202218872.
5. Liu, Y.; Hu, J.; Lu, Q.; Hantusch, M.; Zhang, H.; Qu, Z.; Tang, H.; Dong, H.; Schmidt, O.G.; Holze, R.; et al. Highly enhanced reversibility of a Zn anode by in-situ texturing. *Energy Storage Mater.* **2022**, *47*, 98–104.
6. Chen, Z.; Chen, H.; Che, Y.; Cheng, L.; Zhang, H.; Chen, J.; Xie, F.; Wang, N.; Jin, Y.; Meng, H. Arginine cations inhibiting charge accumulation of dendrites and boosting Zn metal reversibility in aqueous rechargeable batteries. *ACS Sustain. Chem. Eng.* **2021**, *9*, 6855–6863.
7. Li, D.; Cao, L.; Deng, T.; Liu, S.; Wang, C. Design of a solid electrolyte interphase for aqueous Zn batteries. *Angew. Chem. Int. Ed.* **2021**, *60*, 13035–13041.

8. Cao, H.; Huang, X.; Liu, Y.; Hu, Q.; Zheng, Q.; Huo, Y.; Xie, F.; Zhao, J.; Lin, D. An efficient electrolyte additive of tetramethylammonium sulfate hydrate for dendritic-free zinc anode for aqueous zinc-ion batteries. *J. Colloid Interface Sci.* **2022**, *627*, 367–374.
9. Zhang, W.; Dong, M.; Jiang, K.; Yang, D.; Tan, X.; Zhai, S.; Feng, R.; Chen, N.; King, G.; Zhang, H.; et al. Self-repairing interphase reconstructed in each cycle for highly reversible aqueous zinc batteries. *Nat. Commun.* **2022**, *13*, 5348.
10. Hao, J.; Yuan, L.; Zhu, Y.; Jaroniec, M.; Qiao, S.-Z. Triple-function electrolyte regulation toward advanced aqueous Zn-ion batteries. *Adv. Mater.* **2022**, *34*, 2206963.
11. Wu, H.; Yan, W.; Xing, Y.; Li, L.; Liu, J.; Li, L.; Huang, P.; Lai, C.; Wang, C.; Chen, W.; et al. Tailoring the interfacial electric field using silicon nanoparticles for stable zinc-ion batteries. *Adv. Funct. Mater.* **2023**, *23*, 2213882.
12. Zhao, F.; Jing, Z.; Guo, X.; Li, J.; Dong, H.; Tan, Y.; Liu, L.; Zhou, Y.; Owen, R.; Shearing, P.R.; et al. Trace amounts of fluorinated surfactant additives enable high performance zinc-ion batteries. *Energy Storage Mater.* **2022**, *53*, 638–645.
13. Zhou, M.; Chen, H.; Chen, Z.; Hu, Z.; Wang, N.; Jin, Y.; Yu, X.; Meng, H. Nonionic surfactant coconut diethanol amide inhibits the growth of zinc dendrites for more stable zinc-ion batteries. *ACS Appl. Energy Mater.* **2022**, *5*, 7590–7599.
